# Ixekizumab May Improve Renal Function in Psoriasis

**DOI:** 10.3390/healthcare9050543

**Published:** 2021-05-07

**Authors:** Giuseppe Fabrizio Amoruso, Steven Paul Nisticò, Luigi Iannone, Emilio Russo, Giuseppe Rago, Cataldo Patruno, Luigi Bennardo

**Affiliations:** 1 Unit of Dermatology, Mariano Santo Hospital, 87100 Cosenza, Italy; fabrizio_amoruso@hotmail.com; 2 Unit of Dermatology, Department of Health Sciences, Magna Graecia University, Viale Europa SNC, 88100 Catanzaro, Italy; iannone02@yahoo.it (L.I.); erusso@unicz.it (E.R.); cataldopatruno@libero.it (C.P.); luigibennardo10@gmail.com (L.B.); 3 COVID Pneumology Unit, Ospedale di Rossano, 87067 Rossano, Italy; ragogf@gmail.com

**Keywords:** ixekizumab, psoriasis, chronic renal failure

## Abstract

Background: Psoriasis is a chronic dermatological condition characterized by lesions on extensor surfaces, hands, feet, and genital areas. Chronic renal failure is often associated with metabolic syndrome and inflammatory conditions, such as psoriasis. Case report: In this paper, we report a patient with stage-three chronic renal failure that improved his renal condition after treatment with ixekizumab, an anti-IL17A drug used in the treatment of various cutaneous and rheumatological conditions. Conclusions: IL17A blockage may help to treat various autoimmune and inflammatory conditions, such as psoriasis, that may lead to renal impairment. Further investigation is necessary in order to prove the effectiveness of this drug in renal conditions.

## 1. Introduction

Psoriasis is a chronic dermatosis characterized by scaly erythematous patches found prominently on extensor surfaces [1]. Other areas can also be affected, such as hands, feet, or genital areas [2]. Inverse or flexural psoriasis is characterized by the involvement of folds and flexor surfaces, such as the genital area, the submammary and subaxillary regions, the ears. The involvement of hands and feet characterizes palmoplantar psoriasis. Renal impairment is often related to metabolic problems and autoimmune diseases such as psoriasis [3]. Patients affected by psoriasis have an augmented risk of developing chronic kidney disease and, consequently, end-stage renal disease. Additionally, patients with renal problems have a higher risk of developing psoriasis [4]. The severity of renal impairment is usually linked with the psoriatic condition’s severity [5]. Various treatments have been proposed for the management of this condition. Topical drugs such as vitamin D derivatives and corticosteroids manage mild to moderate forms [6]. For more severe forms, systemic treatments such as cyclosporin, apremilast, dimethyl fumarate, or methotrexate were the drugs of choice [7,8,9,10]. In the last couple of years, biological treatments have been proposed to manage various inflammatory conditions, such as inflammatory bowel disease or psoriasis [11]. These drugs target and block selectively various cytokines, such as tumor necrosis factor (TNF) alpha, interleukin (IL) 17 and 23 and guarantee a higher success rate in the management of the condition than traditional systemic treatments [12,13]. Ixekizumab is an anti-IL17 drug approved for the treatment of moderate to severe psoriasis. In this paper, we report the case of a critical improvement of renal function in a patient who has psoriasis and stage-three chronic renal failure.

## 2. Case Description

**Figure 1:**
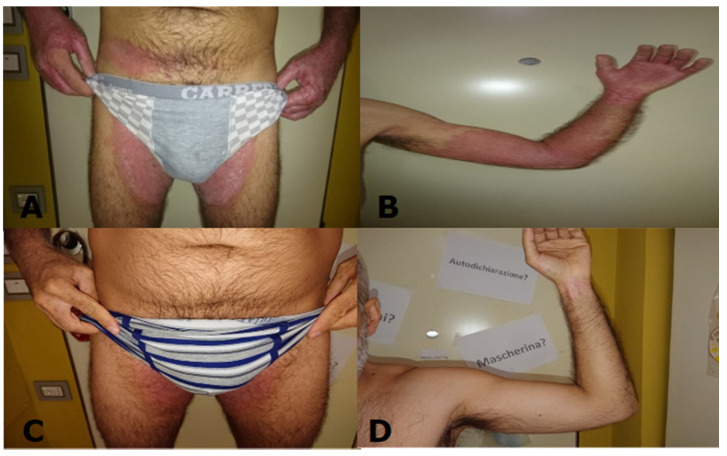
(**A**) Patient at baseline genital area; (**B**) patient at baseline left arm; (**C**) patient after one year, genital area; (**D**) patient after one year, left arm.

A 44-year-old male with a diagnosis of psoriasis since adolescence and chronic mild nephropathy since 2016 came to our attention seeking medical treatment. The patient was previously treated with topical and systemic steroids, cyclosporin A, adalimumab, and phototherapy. None of these therapies led to a constant improvement of cutaneous manifestations. During the dermatological evaluation, psoriasis with an inverse and a palmoplantar component was evidenced (Figure 1). A Psoriasis Area Severity Index (PASI) of 15 was calculated. The patient underwent nephrological consultation for a stage-three renal failure, with a glomerular filtration rate (GFR) of 50 mL/min. Before starting any systemic treatment, a complete panel of blood exams (blood count, ALT, AST, HBSAB, anti-HCV, creatinine, total cholesterol, glycemia, gamma gt, serum protein electrophoresis, ana, ena screen, Interferon Gamma Release Assay, etc.) was performed. The results of this panel were unremarkable except for creatininemia, which was 1.8 mg/dL.

A treatment with anti-interleukin 17 Ixekizumab (160 mg at week 0, followed by 80 mg at Weeks 2, 4, 6, 8, 10, and 12, then 80 mg every 4 weeks) to treat psoriasis was started.

During the first follow-up visit, three months after the beginning of treatment, cutaneous lesions were dramatically improved (PASI 5). After six months, another complete blood panel was performed. Creatinine dropped to 1,0 mg/dL, and therefore a GFR exam was performed; results showed a filtration rate of 95 mL/min, corresponding to a stage-one renal failure. After one year of anti-interleukin 17 therapy, at the third follow-up visit, the patient’s skin clearance was almost complete (PASI 1), and creatininemia was still 1.0mg/dL (Figure 1).

## 3. Discussion

The effectiveness of biological drugs in stabilizing renal function in patients treated for rheumatoid arthritis was recently proved in a study. Patients receiving anti-TNF alpha therapy had a better GFR over time than patients not receiving biological treatments. The researchers hypothesized that these results were related to the drug’s anti-inflammatory effect, as renal failure is usually associated with systemic inflammation [14].

A recent retrospective study on 32 patients with renal failure treated for psoriasis with different biologic drugs evaluated this issue. These patients, all characterized by a GFR inferior to 60 mL/min, were followed during their biological treatment. Although the results showed no statistically significant differences in mean GFR before and after treatment, eight patients showed remission of the GFR. One of these was treated with ixekizumab. The other patients treated that showed remission were treated with infliximab (four patients), ustekinumab (two patients), and adalimumab (one patient). All GFR improvements ranged from 1 to 46%, and the patient treated with ixekizumab registered an improvement of 7% [15]. Similarly, in the case we describe that a psoriatic patient with renal failure treated with ixekizumab showed a dramatic renal function improvement. However, differently from the first report, the GFR improvement was consistent (increasing from 50 to 95 mL/min, almost 100%). Surprisingly, previous reports describe only a 50% improvement in GFR in a patient treated with infliximab as the most consistent improvement [15].

IL-17A is an essential component of the IL-17 cytokines’ family, together with IL-17B, C, D, E, and F. IL-17A is a pleiotropic molecule generating proinflammatory effects acting in synergy with other inflammatory mediators such as IL-1-β and TNF [16].

Summers et al. proved that the in vivo delivery of Th17 cells into mice resulted in the development of albuminuria, glomerular neutrophil infiltration, and increased renal CXCL1 mRNA levels [17]. Various preclinical studies investigating IL-17A-deficient mice or neutralizing anti-IL-17A antibodies demonstrated protective effects in experimental renal diseases [16].

Ixekizumab is a humanized antibody that binds anti-interleukin 17A (IL-17A), neutralizing the inflammation derived from this molecule [18]. Humanized antibodies carry a lower risk of inducing immune responses in comparison to chimeric or mouse antibodies [19]. Experimental papers highlight a potential role of THelper (TH)17-type (IL-17A and IL23) cytokines, although their production source is unclear, in the pathogenesis of various renal disorders, such as lupus nephritis [20,21]. IL-17A has emerged as a promising therapeutic target in immune and chronic inflammatory diseases, including hypertension and chronic kidney diseases. Elevated circulating IL-17A levels have been reported in patients with hypertension and associated conditions, such as systemic lupus, chronic allograft rejection, and preeclampsia [22]. Interestingly, the injection of IL-17A originated an increased systolic blood pressure in mice linked to endothelial and vascular damage [23].

In renal biopsies of patients with a histopathological diagnosis of hypertensive nephroangiosclerosis, IL-17A-positive cells were found mainly in areas of focal inflammatory cell infiltration [24].

IL-17A targeting decreased kidney inflammation in preclinical renal damage models, suggesting that ixekizumab could be an anti-inflammatory treatment to prevent hypertension-induced renal inflammation [25].

IL-17F has been described as a circulating inflammatory molecule linked with an augmented risk of renal damage progression. Interestingly, circulating IL-17A levels are linked to the gravity of CKD and progressively decrease from subjects with low levels of blood sugar to subjects with type 2 diabetes with and without diabetic nephropathy, highlighting once more the tight connection between IL17 and inflammation and the possible effectiveness of anti-IL17 drugs also in diabetic nephropathy [26].

In this regard, blocking the Th17/IL-17A axis has been suggested as a novel treatment for inflammatory kidney diseases. Beneficial effects of IL-17A blockade were described in preclinical immune- and nonimmune-mediated renal damage [27].

In our case, we think that reduction in the renal failure stage was due to the effectiveness of ixekizumab on the inhibition of the interleukin 17-induced inflammation axis.

## 4. Conclusions

This case and the literature findings indicate that IL17A blockage may help treat various autoimmune and inflammatory conditions that lead to renal failure [28].

Limitations of this case are related to the fact that no renal biopsy was performed to better assess the nephrologic condition on the basis of renal impairment. A biopsy would have been helpful, as an increase in renal function could easily be related only to a subset of nephrologic conditions. Considering the different renal conditions driving the progression of renal failure, further prospective studies may be necessary to prove the efficacy of this drug in renal impairment.

However, given the promising results obtained in this case, we believe that anti-IL17 should be the drug of choice in patients affected by psoriasis and renal failure, and the use of drugs targeting IL17A (such as ixekizumab) should be tested in prospective clinical trials to control hypertension-related secondary conditions.

## Data Availability

The data presented in this study are available on request from the corresponding author. The data are not publicly available due to privacy.
